# Perspective Insights to Bio-Nanomaterials for the Treatment of Neurological Disorders

**DOI:** 10.3389/fbioe.2021.724158

**Published:** 2021-10-12

**Authors:** Johra Khan, Mithun Rudrapal, Eijaz Ahmed Bhat, Ahmad Ali, Mohammad Alaidarous, Bader Alshehri, Saeed Banwas, Randa Ismail, Chukwuebuka Egbuna

**Affiliations:** ^1^ Department of Medical Laboratory Sciences, College of Applied Medical Sciences, Majmaah University, Al Majmaah, Saudi Arabia; ^2^ Health and Basic Sciences Research Center, Majmaah University, Majmaah, Saudi Arabia; ^3^ Rasiklal M. Dhariwal Institute of Pharmaceutical Education & Research, Pune, India; ^4^ Department of Biological Sciences and Bioengineering, Indian Institute of Technology, Kanpur, India; ^5^ Department of Life Sciences, University of Mumbai, Mumbai, India; ^6^ Department of Biomedical Sciences, Oregon State University, Corvallis, OR, United States; ^7^ World Bank Africa Centre of Excellence in Public Health and Toxicological Research (PUTOR), University of Port Harcourt, Port Harcourt, Nigeria; ^8^ Department of Biochemistry, University of Port Harcourt, Port Harcourt, Nigeria

**Keywords:** biomaterials, central nervous system, neuro-imaging, neuro-sensing, drug delivery, neural-tissue engineering

## Abstract

The significance of biomaterials is well appreciated in nanotechnology, and its use has resulted in major advances in biomedical sciences. Although, currently, very little data is available on the clinical trial studies for treatment of neurological conditions, numerous promising advancements have been reported in drug delivery and regenerative therapies which can be applied in clinical practice. Among the commonly reported biomaterials in literature, the self-assembling peptides and hydrogels have been recognized as the most potential candidate for treatment of common neurological conditions such as Alzheimer’s, Parkinson’s, spinal cord injury, stroke and tumors. The hydrogels, specifically, offer advantages like flexibility and porosity, and mimics the properties of the extracellular matrix of the central nervous system. These factors make them an ideal scaffold for drug delivery through the blood-brain barrier and tissue regeneration (using stem cells). Thus, the use of biomaterials as suitable matrix for therapeutic purposes has emerged as a promising area of neurosciences. In this review, we describe the application of biomaterials, and the current advances, in treatment of statistically common neurological disorders.

## Introduction

The Central Nervous System (CNS) is the most complex organ in the human body with little capacity to regenerate. Since tissue regeneration is a prerequisite for reversing the clinical symptoms of neurological disorders, it leaves us with very limited treatment options. Thus, we currently face tremendous challenges in the development of successful therapeutic strategies, and can merely delay the progression of neurological disorders (Kanwar et al., 2012). Theoretically, we can overcome these challenges by cell-based therapies which induce cellular regeneration by delivering growth factors, hormones, neuroprotective proteins and several other bioactive molecules into the CNS. Alternatively, transplantation of stem cells (neural, pluripotent or mesenchymal) can also induce and promote regeneration of damaged neuronal cells by secreting growth factors (Ghuman and Modo, 2016). Practically, the meninges and the blood-brain barrier provide neuro-protection by strictly regulating the movement of the matrix components around the CNS. These regulatory mechanisms become increasingly uncongenial and further limit the diffusion of foreign agents into the CNS, in cases of traumatic injuries and formed lesion sites during advanced neurological disorders. During stem cell therapy, this results in inadequate cellular integration with host tissues, poor cell survival, and unregulated cellular differentiation (Tam et al., 2014; Liu et al.,2021). Efforts to treat these disorders with drug delivery systems face issues like the need for high doses of therapeutics to ensure sufficient diffusion through the blood-brain barrier. Since bolus injections result in uneven or limited exposure to drugs, most often the delivery is done using non-ideal techniques like prolonged infusion and highly invasive intra-ventricular injections among others (Weiss et al., 2009; Rossi et al., 2013). These techniques can result in infections due to long term exposure to mini-pumps (used for drug infusion) and tissue injury respectively (Nau et al., 2010; Parkinson foundation, 2021). For these reasons, the standard treatment strategies have shown poor outcomes in clinical trial studies (Ma et al., 2012; Lemmens et al., 2013).

Despite being discouraging, each unsuccessful clinical trial study has aided us in recognizing the barriers to successful treatment of neurological disorders. We know that the ideal agent must be able to cross the blood-brain barrier and achieve therapeutic levels in the brain. Also, it should ensure safety for long term usage because integration of stem cells with host cells and multiplication is a slow process (Budd Haeberlein and Harris, 2015; Sudhakar and Richardson, 2019). Further, it should be able to enhance stem cell survival (long enough to facilitate integration with host tissue) without triggering an inflammatory response (Gendelman et al., 2015). Besides, the successful treatment strategies also require early diagnosis of disease and identification of the effect of immunogenic factors on the differentiation and behavior of stem cells. The study of the latter parameter has turned out to be critical due to the lack of correlation observed between the *in-vitro* and *in-vivo* behavior of stem cells (Carradori et al., 2017). Considering these factors, the study of biomaterials for treatment purpose is emerging as an important field of neurosciences. This review describes the characteristics of potential biomaterials and the reported progress in treatment of neurological disorders associated with them.

## Characteristics of Bio Nanomaterials Used for Treatment of Neurological Disorders

**FIGURE 1:**
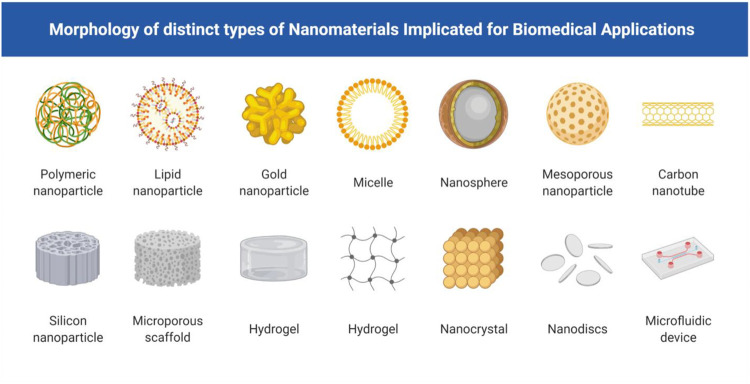
Morphology of distinct types of nanomaterials implicated for biomedical applications including nanoparticles of size range between 1 and 100 nm, which includes hydrogels, Self–assembling peptides, Nano carriers, Liposomes, nanoparticles for therapy and diagnosis of AD.

Various inert and sterilizable biomaterials of natural as well as synthetic origin have been identified as a potential tool for the treatment of neurological disorders. Common examples include cell-free scaffolds, hydrogels, nanoparticles, self-assembled peptides and liposomes, and nanofibers. The different morphological forms of biomaterials are represented in Figure 1. They share the common characteristics of biofunctionality, biocompatibility and biodegradability. They differ in their physical, chemical, biological and mechanical properties (Dos Santos et al., 2017). These properties help in specific characterization of biomaterials for various applications. The biomaterials provide a physical scaffold that allows sustained drug release without the use of invasive procedures and prevents the degradation of therapeutics after delivery (Hoare and Kohane, 2008). They also support stem cells and improve the rate of cell survival (Bordoni et al., 2020). At present, studies based on several *in vivo* animal models have been published with encouraging results. However, several factors including immune- and hemo-compatibility, pyrogenicity and chemical properties of these biomaterials limit their application in clinical studies involving human volunteers (Wang et al., 2004; Masaeli et al., 2019).

### Hydrogels

As the name suggests, hydrogels are hydrated and flexible matrix made of water insoluble polymers that are cross-linked by chemical or physical bonds. They have a strong three dimensional and porous structure with a soft physical appearance that prevents any internal damage to the CNS. Hydrogels physical properties, such as softness and porosity, are affected by changes in pH, temperature, and osmotic pressure. As a result, they’re known as smart biomaterials, and they’re used to regulate the diffusion of nutrients, oxygen, and therapeutics based on the location of the lesion and the level of treatment needed. Examples of natural hydrogels include hyaluronic acid, xyloglucan, chitosan, collagen, sodium alginate, gelatin and protein (silk, lignin, cellulose and keratin) based hydrogels (Kaczmarek et al., 2020). Similarly, it can also be prepared synthetically using polyacrylamide and polyethylene glycol which are non-biodegradable but highly bio-compatible agents. Most commonly either natural or synthetic materials are used in preparation of hydrogels. However, few researchers have reported using a mixture (in varied proportions) of natural and synthetic hydrogels to improve their characteristics for neural applications (SilvaAdaya et al., 2017). Hydrogels can be characterized based on their appearance (microsphere, matrix or film), number and type of polymeric units (homo-, co- and multi-polymeric), charge (nonionic, ionic, amphoteric, electrolyte, zwitterionic), physical appearance (matrix, film, or microsphere) and crystallinity (amorphous, semi crystalline, and crystalline). Besides, they are also classified based on internal bonds and cross-linking structures into polymeric covalently cross-linked hydrogels and self-assembled hydrogels. The former hydrogels are stiff and require surgical implantation whereas the latter preparations are injectable where they self-assemble into hydrogels due to changes in internal physicochemical parameters (Tao et al., 2020).

Hydrogels have properties similar to the extracellular matrix of the CNS, allowing for optimal diffusion through the blood-brain barrier, resulting in effective drug delivery systems (Bordoni et al., 2020). However, hydrogels are mainly used in regenerative treatment because they provide an optimum scaffold for supporting the stem cells before they are completely implanted into the soft tissues. Moreover, they do not disrupt the physical structure of tissues due to their efficient mechanical properties and high water content. Precisely, the stem cells can be encapsulated in the hydrogels prior to implantation (Willerth, 2018). The hydrogels are also capable of infiltrating the neighboring cells by surface expansion. This facilitates oxygen diffusion and waste removal through hydrogels. It also prevents the triggering of severe immune responses in most cases, or acts as a barrier for host immune factors to cause any adverse effect to the implanted cells (Fernandez-Serra et al., 2020).

Numerous approaches have been considered for drug delivery and treatment of neurological treatments. In a recently published study, Ghozalidah et al. (2019), reported optimization of physicochemical and rheological properties of thermo sensitive chitosan-based self-assembling hydrogels to generate aerosol for nasal delivery in treatment of neurological disorders. *In vivo* regeneration studies carried out in rat models has further indicated the potential of peptide-modified gellan gum hydrogels for treatment of spinal cord injuries. In this study, the adipose stem cells co-cultured with olfactory unsheathing cells were encapsulated in the above hydrogel. On application, it showed significant improvement in motor skills and histological parameters of rat models (Gomes et al., 2016). In general, hydrogel regeneration therapies have shown axonal sprouting and upregulation of neural progenitor cells. Moreover, they attenuate the inflammatory response of macrophages and other immune cells and stimulate synapses to rewire the functioning of the neural cells in the region around the lesions (Cook et al., 2017; Nih et al., 2017). In fact, the hydrogel components like hyaluronic acids and gellan gum have been reported to improve neuronal differentiation in stem cells irrespective of the additives used in the preparations (Moshayedi et al., 2016; Koivisto et al., 2017). The studies have also reported improvement in the efficacy of anti-oxidant and anti-inflammatory drugs like Edaravone, through hydrogel based regeneration therapies, in patients who suffered from strokes (Gonzalez-Nieto et al., 2018; Tamer et al., 2018).

### Self-Assembling Peptides and Nano Carriers

Self-assembling peptides (SAPs) are a type of hydrogels that consists of repeating units of amino acids. They form double beta sheets on dissolution in water due to the presence of alternating polar and non-polar residues of amino acids. The peptides form well-defined structures and hence can be used as building blocks for more sophisticated bio-engineering of scaffolds with customized amino acid sequences (Yokoi and Kinoshita, 2012). This approach helps in incorporating novel characteristics of cell adhesion, anti-microbial activity and other useful properties into the peptides to enhance their potential in regenerative medicine and drug delivery systems (Merrifield, 1963). With the advancements in bio-engineering techniques, SAPs have also been constructed using primary sequences which are capable of assembling into higher and more complex nano-carriers (Kiyonaka et al., 2002). The structure and strength of the SAP hydrogels and nano-carriers depend on the chemical bonds between the peptide chains. In addition to amino acids, several monomers and peptides have the ability to assemble into nanostructures like tubes, rods, and sheets (Lee et al., 2019). The alpha-helical structures of SAPs can form nanotubes and nanofibers through hydrophobic and electrostatic bonds. The β-sheets, on the other hand, show gelation properties and form extended β-sheet nanofibers, hence they are a good choice for hydrogel preparations (Mehrban et al., 2015). A common example of peptide sequence is the β-hairpin forming peptides. They are long proline rich sequences that self-assemble into nanofibers with the help of hydrogen bonds (Lewandowska et al., 2010). Single amino acids have also shown gelation properties when capped with aromatic protecting groups (Bowerman et al., 2011). Besides the soluble peptides, the amphiphilic peptides can be used for constructing nano-vesicles, micelles, nano-fibers, and nano-tubes. Also, more stable cylindrical structures can be synthesized using cyclic peptides (Lee et al., 2019).

Similar to the use of biomaterials, nanotechnology is also an emerging field in biological sciences. Their application for medicinal purpose in general and neurosciences in particular, has shown promising outcomes due to their ability to deliver drugs across blood-brain barriers while maintaining their efficacy. They can be synthesized using a vast variety of materials including ceramics (silica), metals (gold and silver), oxides and salts, and polymers (proteins and lipids). Nano-carriers can also be synthesized using biomaterials like protein polymers (collagen) and lipids (micelles). Moreover, they can be prepared in different structural forms like nanoparticles, nanofibers, nanotubes, nanospheres and nanogels. Interestingly, each nano-structure provides a unique property to the drug delivery system. In practical application, the nanofibres have shown tremendous potential in restoration of nerve tissues. Like SAPs, the fibrous structure of nanofibres promotes proliferation of axons in the CNS by attenuating their typical mechanisms.

The SAPS and nano-carriers exhibit variable physicochemical and biochemical properties depending on their size, structure and surface area. In general, it is possible to trigger self-assembly of various peptide sequences to form nano-structures and hydrogels by optimizing various physicochemical parameters like pH, temperature, ionic nature and enzymatic conditions (Nisbet and Williams, 2012). Optimization of these parameters can also help in designing multiple assemblies to form supramolecular networks (Kiyonaka et al., 2004; Maclean et al., 2016), thus opening the door to innumerous possibilities.

The scope of SAPs has been well defined in neurological studies as drug delivery systems and neuronal regeneration therapies. The most promising derivative of SAPs has been recently developed from RADA16 and KLDL12 family as 3D scaffolds for cells (Tran et al., 2020). They reported facilitated regeneration of the injured tissues in the CNS on transplantation of microvascular cells in RADA-16I scaffold. Further analysis indicated the formation of microvessels which encouraged the infiltration of axons, and improved vascularization at lesion sites. The similarity in these SAPs scaffolds and extracellular matrix of CNS holds promising scope for regenerative medicine in treating neurological disorders through improvisation in the SAP properties. For instance, the role of amyloid proteins in neurogenesis and neuron differentiation has been well established in several studies (Gakhar-Koppole et al., 2008; Zhou et al., 2008). Cui et al. (2016) suggested that the functional domain Tyr-Ile-Gly-Ser-Arg (YIGSR) which was derived from laminin protein promoted neuronal differentiation in Alzheimer-rat models. The treatment with SAP also showed significant cognitive and behavioural improvement in rats. It has been proposed that the assembly of functional proteins in biomaterial scaffolds can improve cell adhesion, proliferation, differentiation, and maturation (Zhao et al., 2008). The SAPs prepared with amphiphilic molecules can be used as a suitable medium to carry growth factors and hormones required to promote regeneration at the lesion sites of neurological disorders. A recently published study proposed regenerative therapy using RADA16-SVVYGLR peptide that self assembles into nanofibers. The *in vivo* zebra-fish model showed neural stem proliferation, sprouting angiogenesis and developmental neurogenesis on application of the nanopeptide hydrogel. Moreover, the physical properties of the hydrogel were reported to be programmable which enhanced the potential of regenerative ability of injured tissues (Wang et al., 2017). Table 1 summarizes the different nanoparticles and there use in neurology.

**TABLE 1:** Summarization of different metal core nanomaterials for different functions in Neurological diagnostics.

Nanomaterial metal core	Type of nanoparticles	Route of administration	Modelin vivo/*in Vitro*	Mode of action	References
Iron	Ferumoxtran or Ferumoxytol	Intravenous	*In vivo*	Help in diagnosis of cellular imaging of human ischemic stroke	Yang et al. (2016)
	Endorem	Intravenous or Intracerebral	*In vivo*	Help in stem cell movement and growth in central nervous system and as contrasting agent for nodal stages. It remains unaffected by metastatic cells	Sonawane et al. (2019)
	Chitosan	Exposition	*In vitro*	Useful in determination of acetylcholine in synthetic urine	Shcharbina et al. (2013)
Gold	Quercetin	Intravenous	*In vivo*	It promotes the fusion of autophagosomes and lysosomes. Helps in cleaning Aβ -induced cytotoxic damage	Wong et al. (2012)
	Clioquinol	Intravenous	*In vivo*	These nanoparticles inhibit β40 plaques, aggregation, cell membrane disruption, and ROS- mediated apoptosis	Kaushik et al. (2018)
Silver	Cellular prion protein 95-110	Incubation/Intravenous	*In vivo*	Useful in detection of Aβ oligomer with a peptide as the bioreceptor	Liu et al. (2020)
	Gadolinium	Intravenous	*In vivo*	Used as contrast agent for magnetic resonance imaging	Overk at el., 2014

## Other Potential Bio Nanomaterials

**FIGURE 2:**
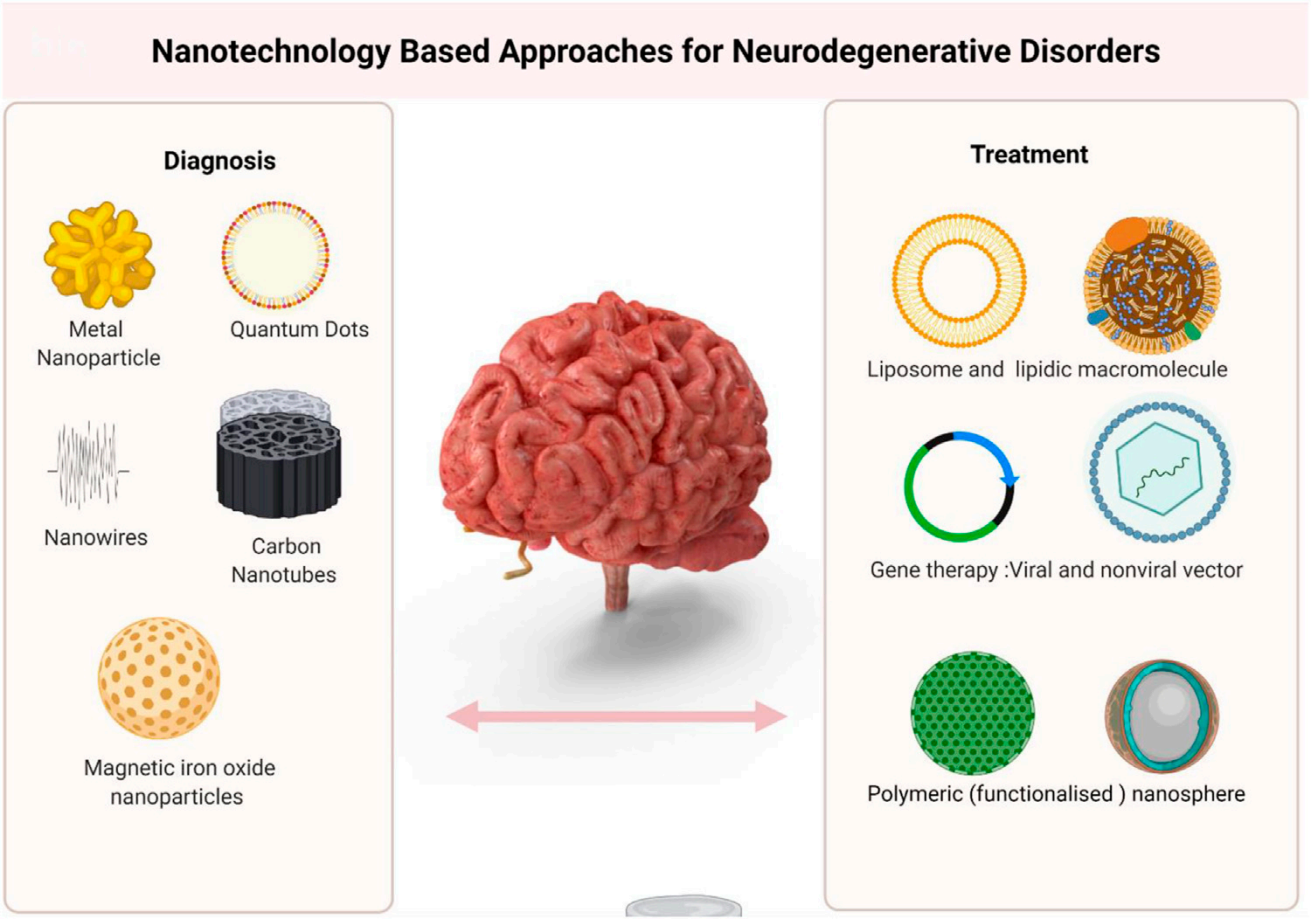
Different form of NPs and their Application to treat and control Neurological Disorder.

The hydrogels, SAPs and nanocarriers are the most powerful biomaterials which have shown tremendous potential in treatment of neurological disorders such as Alzheimer diseases, Parkinson’s disease, stroke, spinal cord injury and tumors (Figure 2). Undoubtedly, hydrogels have been identified as a potential candidate for neuro-regenerative approaches in treatment of above mentioned neurological disorders and many more. Besides, few uncommon biomaterials are also reported in literature. One example of uncommon biomaterials is cell free scaffolds. They are biodegradable scaffolds which can be obtained using regenerating tissues by processes like electro spinning, freeze-drying and even 3-D printing. A major drawback of the use of cell free scaffolds as biomaterials is higher degree of hydrolysis and enzymatic degradation. However, considering their tissue origin, they are highly compatible and trigger minimal side effects on transplantation. Like other biomaterials, the mechanical properties can be monitored and adjusted based on the extent of lesions in neurological disorder.

The functional biomaterial is another interesting candidate in this list. They are known as stimuli-sensitive nanomaterials which respond to endogenous (pH gradient, redox processes, enzymes and other biomolecules) or external stimuli (like magnetic and electric fields, ultrasound, temperature and light). Hence, they enable non-invasive neuro-imaging potential that can significantly facilitate the diagnosis of neurological diseases. This, in turn, can improve the treatment outcome considerably. Moreover, being biocompatible and biodegradable, they can be hydrolyzed after used and flushed from the system, thus eliminating any chances of infection, inflammation or surgical removal of implants (Shan et al., 2019). Currently, a major drawback of these biomaterials is that the application of external stimuli is challenging for target nerve tissues. However, with guided research, the stimuli-responsive nanomaterials have the potential to provide the cutting edge inspiration and emerge as an effective drug delivery and diagnostic vehicle (Blum et al., 2015). Table 2 provides an overview of advances in biomaterial applications in neurological studies.

**TABLE 2:** Advances in bio nanomaterial applications in neurological studies.

Sr. No.	Biomaterial	Study model	Research outcome	References
1	Magnetic polylactic-co- glycolic acid (PGLA) scaffolds	Middle cerebral artery occlusion (MCAO) Rat models	Neural stem cells supported with a PLGA scaffold reduced the ischemic lesion volume in the cerebral cortex of rat models	Bible et al. (2012)
2	PLGA microcarriers	Ischaemic stroke rat model	Mesenchymal stem cell survival and proliferation	Quittet et al. (2015)
			Effective delivery of vascular endothelial growth factor	
			Increased angiogenesis	
3	Alginate conduit internal filler	50 mm gap sciatic nerve injury Cat model	Nerve regeneration and proliferation. A 50 mm sciatic nerve gap was reduced to 10 mm 7 weeks after surgery	Askarzadeh et al. (2020)
4	3D bioprinted methacrylated gelatin nerve conduits	*In vitro* study	Highly hydrophilic in nature. Can readily bind with nano-carriers and platelets to promote peripheral nerve regeneration	
5	chitosan-polylactic acid (PLA) blend	*In vitro* study	Improves mechanical properties and biocompatibility. Reduced myofibroblast infiltration and scar tissue formation, and physically support regenerating nerves	Xie et al. (2008)
6	Silk fibroin-collagen blend matrix	10 mm gap sciatic nerve defects rat models	Adipose stem cells co-cultured with Schwann cells accelerated nerve regeneration	Xu et al. (2016)
7	Nanoliposomes like dipalmitoyl phosphatidyl choline, dipalmitoyl phosphatidyl serine and ganglioside	Rat models	Improved delivery of citicoline to the brain functional recovery and synaptic plasticity after COVid-19 related brain damage	Saghazadeh and Rezaei, (2021)
8	poly-l-lactic acid and gelatin scaffold	Rat models	Differentiation of neuronal stem cells into motor neurons	Binan et al. (2014)
9	Tri-layered silk scaffold	10 mm gap sciatic nerve defects rat models	No inflammation or scar formation was observed 2 weeks after repair indicating progressive axonal regrowth	Alessandrino et al. (2019)
10	Keratin and alginate scaffold	*In vitro* study	Improved mechanical properties of keratin scaffold. Improved peripheral nerve repair	Gupta and Nayak, (2016)

## Stimuli-Responsive Nano-System

**FIGURE 3:**
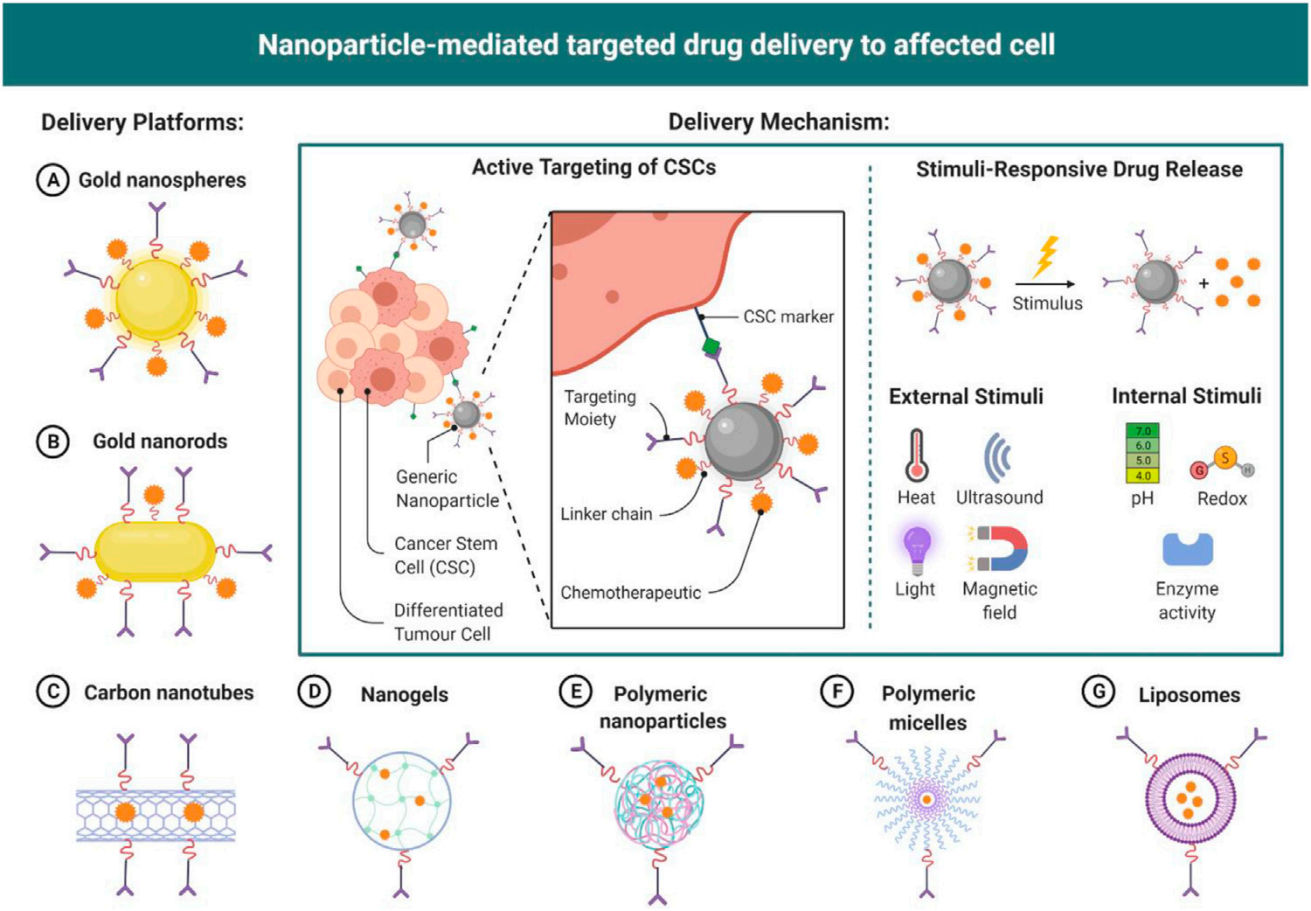
Different nanomaterial and nanomaterial mediated drug delivery to affected cells.

Stimuli responsive bio nanomaterial hold numerous potential in improving drug delivery system, many of them are under clinical trials. Pegylated liposomal doxorubicin (PLD), a nano-assembly is approved by FDA for variety of cancer as it reduces potential side effects (Devnarain, et al., 2021). A nano-assembly includes many important properties against different type of stimuli, one of which is to function as on demand drug delivery system against exogenous (Light, temperature, magnetic fields, and electric fields) and endogenous (pH variation, enzymes, and redox gradients) stimuli. In recent years the development of stimuli response nanocarriers for the use in CNS disease therapy is studied in details (Naqvi et al., 2020). The nanocarriers are divided into three types on the basis of responsive factors which are; external and internal stimuli-responsive nanocarriers, and multifunctional responsive nanocarriers. Stimuli like pH plays important role in CNS lesion area differentiation from other normal tissues (Liu, F et al., 2016). The low pH induced due to lack of nutrients and oxygen creates an acidic microenvironment induces multi-drug resistance. The pH responsive nanocarriers help in drug delivery to these tissues. Similarly enzyme responsive nanocarriers are useful for programmed drug delivery in disease conditions which induce enzymes. Some other stimuli response nano-system is discussed below in different disease conditions with advancement in treatment using nanomaterials (Figure 3). CRISPR mediated drug delivery to tumor cells targeting expression of Cas9 protein using nucleic acids or by ribonucleoprotein complexes as a transportation method is most relevant but with limitations and challenges of with immunogenicity related safety risk. To overcome these limitations pH sensitive nanoparticles filled CRISPR-Cas9 were constructed with poly ethyleneimine-poly lactic -coglycolic acid attached to cleavable linker. Paclitaxel released from these nano particles produce antitumor immunomodulatory effect, resulting in T-cell population regulation, dendritic cell activation, induction of immunogenic cell death, and macrophage repolarization. Shao et al. (2020) produced core-shell nanoparticles targeting immunosuppression, angiogenesis, and uncontrolled proliferation that are major tumor growth factors. Core shell nanoparticles in combination with anti-programmed death ligand antibody (aPDL1) is found to generate regression of primary tumor studied on mouse model.

## Recent Advances in the Treatment of Common Neurological Disorders Using Nanomaterials

### Alzheimer’s Disease

Alzheimer’s is a progressive neurodegenerative disorder characterized by dementia and impaired cognitive functions. Statistically, 6.2 million Americans aged 65 and above are diagnosed with Alzheimer’s disease (Alzheimer’s Association, 2021). It is believed that amyloid-β peptide aggregation and neurofibrillary tangles trigger the pathogenesis of Alzheimer’s disease. Hence, modulation of amyloid-β protein or inhibiting its aggregation, and engineering of neural tissues has been focused mainly on Alzheimer’s treatment strategies (Serrano-Pozo et al., 2011). At present, there is no cure for Alzheimer’s. However, although some side-effects are reported, the available medications can significantly slow down its progression.

Based on clinical evidence that epigallocatechin-gallate (EGCG) acts as an inducer of α-secretase activity, which facilitates proteolysis of amyloid precursor protein (Torre, 2021), Manzine et al. (2019) formulated polyethylene and PLGA nanoparticles loaded with EGCG. They proposed stabilized drug delivery in mice models that accompanied enhanced spatial learning and memory. They further proved the efficacy of above formulation through biochemical markers like increase in synapses, reduction of neuro-inflammation, reduction in amyloid β plaque burden and cortical levels of soluble and insoluble amyloid-β peptide. Similarly, another approach utilized quercetin encapsulated lipid nanoparticles and functionalized them with transferrin to facilitate their passage across the blood-brain barrier (Pinheiro et al., 2020). These biomaterials could easily trap quercetin molecules and showed effective fibril inhibition capacity. Similar studies using polyethylene and lipid formulations with pomegranate extract and curcumin, respectively, have indicated restoration of histological hallmarks of Alzheimer’s pathology affected by neurofibrillary tangles, β-amyloid plaques and Nissl granules in mice models. Neuroprotective activities through increased levels of inflammatory cytokines in prefrontal cortex, hippocampus and serum of mice have also been reported (Giacomeli et al., 2019; Almuhayawi et al., 2020). Also, in general, the cationic biomaterials containing chitosan have shown suppression of oxidative stress in Alzheimer’s disease. In contrast, anionic biomaterials like those containing phosphatidic acid or cardiolipin have shown high affinity for binding to amyloid-β peptide aggregates (Gobbi et al., 2010; Bereczki et al., 2011; Dhas and Mehta, 2020). Additionally, the intracellular aggregation of tau (microtubule-associated protein) into neurofibrillary tangles is also associated with progression of Alzheimer’s. In this respect, a peptide amphiphile SAPs, carrying curcumin, was found to induce tau hyperphosphorylation and increase its clearance *in vitro* experiments with a medullo-blastoma cell line (Altunbas, 2011). Another study by Cui et al. (2015) demonstrated the stability of neuronal stem cells in SAPs matrix containing a functional domain Tyr-Ile-Gly-Ser-Arg (YIGSR) derived from laminin in cell replacement therapy. The neuronal stem cells transplantation in Alzheimer rat models demonstrated improved cognitive behavior with restoration of memory functions. The apoptosis levels in the CA1 region and amyloid-β peptide levels in the hippocampus were also found to decrease on transplantation. Thus, significant progress has been made, with the aid of biomaterials, in managing (or delaying) the progression of the disease. However, there are no significant clinical trials in literature to suggest effective treatment outcomes and ensure complete treatment of Alzheimer’s as yet.

### Parkinson’s Disease

It is another neurodegenerative disorder characterized by bradykinesia (slowing of movement), along with muscle rigidity and tremors due to loss of activity in the substantia nigra pars compacta region. The fibrillar α-synuclein inclusions (Lewy bodies) and dopaminergic neurons are identified as biomarkers to detect progression of Parkinson’s disease. Although other misfolded protein aggregates, ubiquitin–proteasome system (UPS) impairment, mitochondrial dysfunction, oxidative stress and inflammation contributing to dopaminergic cell condition, are also associated with Parkinson’s disease. Hence, the current strategies to treat Parkinson’s mainly include targeting α-synuclein accumulation and supplementing growth factors and other agents (including synthetic dopamine). Secondary mechanisms to overcome oxidative stress, mitochondrial dysfunction and inflammation are also effective in management of the disease symptoms (Yarnall et al., 2012; Dexter and Jenner, 2013). Currently, more than 10 million people are living with Parkinson’s disease, throughout the world (Parkinson foundation, 2021). Like Alzheimer’s, there is currently no effective treatment for Parkinson’s disease and all available medicines, although available, show some side effects on long term use.

With the aid of nanotechnology, chitosan and cellulose scaffolds have shown promising effects in dose-dependent increase and sustained delivery of dopamine (Trapani et al., 2011; Garbayo et al., 2013). These findings are specifically promising since any other improvisation has been unsuccessful in delivering dopamine through the blood-brain barrier. Various studies have also reported the use of hydrogels, collagen and synthetic biomaterials like PGLA in dopamine delivery (Pahuja et al., 2015). Similarly, other anti- α-synuclein agents have also been successfully delivered by encapsulating them in the immunoliposomes (Loureiro et al., 2015). An interesting study by Breydo et al. (2016) reported the use of reversible addition–fragmentation chain transfer (RAFT) polymerization technique to synthesize hyper-branched polyethylene glycol structure containing a dopamine moiety. Although the anti-fibrillation activity of this system was low as compared to free dopamine, this study suggested the possibility of incorporating novel functional groups within polymers and probably achieving better biological activity in near future. The most convincing study reported so far, however, is based on the use of human pluripotent stem cell derived dopaminergic progenitors. In this study, a biomimetic hydrogel matrix was programmed to support dopaminergic progenitors by including a laminin epitope within the matrix and encapsulating glial cell line-derived neurotrophic factor. This approach resulted in 51% increase in A9 neuron cells (a subpopulation of dopaminergic neurons) that overall improved motor deficits in study models. The hydrogel matrix further enabled standardization and predictability that is essential to further customize the study designs (Hunt et al., 2021).

### Spinal Cord Injury

The spinal cord injury can lead to devastating neurological conditions including loss of sensory and motor functions. It is estimated that more than 27 million people are victim to events like motor vehicle accident, fall, sporting injury or an act of violence. Most of these patients sustain spinal cord injury with local cellular damage (primary phase) and more complex cascade of cellular events like ischemia, inflammation, edema, cell death, axonal degeneration, gliosis and formation of scar tissue (secondary phase). Regenerative treatment therapies are the key to successful recovery from spinal cord injury. However, at present the therapies are limited to adaptive and rehabilitative approach (Hagen et al., 2012; Assuncao-Silva et al., 2015).

In comparison with other neurological disorders, the use of biomaterials is more common in treatment of spinal cord injury. This is because the infiltration of glial cells and oligodendrocytes as a part of natural defense mechanism against scarring, and later their apopotosis, forms cystic cavities that prevent axonal regrowth and neurite outgrowth. This severely narrows the possible treatment and complete recovery of patient using standard approaches. Also, the cystic cavities in spinal injury sites present a major obstacle (besides blood-brain barrier) in drug delivery. To overcome this major hurdle, an intrathecal delivery of a polymeric nanocomposite hydrogel was attempted by Baumann et al. (2010) as a minimally invasive approach for 28-day sustained release of drug in rat models. Although, regaining of significant locomotor functions were not observed in this study, they indicated efficacy of hydrogel as therapeutic model, based on absence of inflammation, scarring or cavity volume as compared to controls. This was also among the first studies to confirm the safety of biomaterials on month long exposure. As a result of further development in this area, the extracellular matrix remodeling was suggested in a recent study to stimulate tissue repair in events of spinal cord injury. The study demonstrated a unique and dynamic interaction between inflammatory cells (like macrophages) and a thermosensitive imidazole-poly (organophosphazene) hydrogel which was capable of eliminating cystic cavities in rat models (Hong et al., 2017). Interestingly, according to the most recent study, Taxol (a clinically approved antitumor drug) loaded biological functionalized SAPs nanofiber scaffold can promote neurite extension, prevent inflammation and demyelination of neurons, and control cavity dimensions in spinal cord injury. Hence, it is an excellent model and therapeutic strategy to promote recovery in spinal cord injury (Xiao et al., 2020). Another interesting and promising approach is fabrication of tissue-like constructs for treatment of spinal cord injuries. However, tissue engineering faces several practical and ethical limitations in clinical studies. An *in-vitro* 3D model of hydrogel with functional neuronal network of human neural stem cells, however, has shown active proliferation, differentiation, and maturation of stem cells into desired neurons and improved behavioral recovery. Although promising outcomes were not observed in terms of electrical activity of neural networks, this attempt certainly opens opportunities for novel strategies in treatment of neurological disorders (Marchini et al., 2019).

### Stroke

Stroke occurs due to a cascade of pathological biochemical reactions, following interruption in blood circulation which leads to deprivation of oxygen and nutrients in brain cells and tissues. It can be caused due to blockage in artery, tumors or internal bleeding, and invariably results in disability or death. The occurrence of haemorrhagic stroke (due to internal bleeding) is a rare occurrence with fatal outcome. However, the ischemic stroke (resulting from interruption in blood supply) is commonly observed in stroke victims. At present, ischemic stroke is the second leading cause of death and disability, globally.

The establishment of reperfusion is theoretically achievable in reversing the ischemic stroke. However, practical evidence has indicated significant nerve damage at the sight of stroke, in a very short period of time. Subsequently, the neighboring tissues are also affected due to the series of pathological events like oxidative stress, depolarization, inflammation, and excitotoxicity that follows in a short time after occurrence of stroke. The major application of nano-materials in stroke treatment includes enhanced permeability to blood-brain barrier and controlled drug release to stop the bleeding (Molina and Alvarez-Sabin, 2009; Moskowitz et al., 2010; Zhan and Lu, 2012). Nano oxygen carriers are another application of nano materials to aid in oxygen supply through liposome-encapsulated hemoglobin. These systems are similar in structure and function to red blood cells, and also prevent the risk of hemorrhage (Kawaguchi et al., 2010). However, nerve regeneration is mostly targeted in advanced research to replace damaged neuronal nerves and regain muscular and motor ability. An early study provided evidence that antibody against Nogo-R (myelin-derived axonal outgrowth inhibitor) can be stabilized in hyaluronic acid hydrogel. The study further demonstrated neuronal regeneration and promotion of functional recovery in animal models of stroke (Ma et al., 2007). In a recent approach, a three-dimensional self-assembling peptide nanofiber hydrogel was functionalized with laminin-derived motif (IKV) as well as brain-derived neurotrophic factor (RGI) for peripheral nerve regeneration in male Sprague-Dawley rat models. This study not only demonstrated the nerve repair, myelination and enhanced gene expression of various neural growth factors, but also confirmed a synergetic activity of IKV and RGI on axonal regrowth and function recovery after peripheral nerve injury (Yang et al., 2020).

The use of biomaterials has thus contributed to considerable progress in the treatment of common neurological disorders. Although all studies do not show ideal outcomes against the proposed hypothesis, they certainly reduce the gaps in knowledge, and provide an increased understanding of the pathophysiology of neurological disorders, giving a way to improvised interventions. The application of biomaterials is certainly a futuristic approach, and unless a magically programmable or tissue-like engineered scaffold is introduced, finding a substitute may be challenging (Figure 3).

## Advantages of Nanomaterials for Central Nervous System Applications

The common advantages of various nanomaterials include enhanced bioavailability, sustained drug release, protection from degradation of compounds and most importantly, the simultaneous delivery of multiple agents. The accuracy, specificity and sensitivity offered by nanotechnology can immensely help in detecting even low levels of biomarkers associated with neurological diseases. Hence, it has extensive application in diagnostics. Unlike other treatment strategies, the molecular interaction between cells and nano-materials allow targeting the damaged cells or lesion sites for drug delivery. Also, this characteristic has been exploited tremendously to target dopaminergic neurons and amyloid-β peptide aggregates for treatment of Parkinson’s and Alzheimer’s disease respectively. Theranostic based nanotechnology (use of radioactive drugs for diagnosis and therapy) further widens the window of possible applications in precision drug delivery systems. Nanomaterials are the only agent through which minimal doses of medicines can be effectively used for achieving best outcomes (Zhong and Bellamkonda, 2008; Niemczyk et al., 2018; Jarrin et al., 2021). The advantages of the use of biomaterials in treatment of neurological disorders can be summarized in the following points.

• Biomaterials are ideal for crossing the blood-brain barrier BBB and hence present futuristic diagnostic and treatment potential for neurological disorders.
• Most importantly, the excellent biocompatibility shown by biomaterials prevents the chances of toxicity, which is currently a major limitation with synthetic nano-materials.
• Hydrogels, in particular, are attractive scaffolds for tissue regeneration due to their mechanical stability. In addition, the *in situ* gelling formulations are ideal alternatives to invasive implantation surgeries that would otherwise require suitably designed scaffold of specific dimensions to fit irregular lesion cavities.
• Hydrogels are highly porous and hence till now there is no superior alternative for drug carrier system or stem cell binding scaffolds that can diffuse through the blood-brain barrier.

## Limitations and Future Approaches of Nanomaterials for Central Nervous System Applications

Apart from common limitations of nano-materials like varied solubility at different temperatures, and toxicity of synthetic materials (due to high surface area leading to high reactivity), the cost of nano-medicines and nano-techniques are the biggest barrier in application of nanotechnology. Also, although most studies have described biomaterials to be highly compatible, the inflammation or oxidative stress triggered on their application cannot be predicted. Additionally, the factors like biomaterial diffusion in non-specific neural areas that may trigger unexpected biochemical reaction cannot be overlooked. Besides, the phenomenon of ‘burst release’ of drugs/proteins into the matrix of CNS is currently a big challenge. In simple terms, it is the likelihood of diffusion of higher (than desired) concentration of therapeutic agents at the time of incorporation of hydrogels at specific sites. Although, it can be overcome by cross-linking of therapeutic agents or use of high-affinity ligands, the prediction and design of absolute systems remains a challenge (Soontornworajit et al., 2010; Lin et al., 2021).

However, in spite of the above limitations, it is also important to note that biomaterials are the most practical alternatives to existing treatment strategies available for neurological disorders. Also, the currently faced limitations can be used as a basis for further research and development in the field of nanotechnology. Most importantly, any existing strategy has their own set of advantages and limitations. It is the choice of combined therapies that are unique in a given medical scenario, which truly challenges the expertise of a clinical practitioner.
